# Supplementary material for: Sociocultural and patient-health care professional related factors influencing self-management of multiethnic patients with multimorbidities: A thematic synthesis
Source: PLOS Glob Public Health. 2023 Sep 21;3(9):e0002132. doi: 10.1371/journal.pgph.0002132 (PMC10513255; doi:10.1371/journal.pgph.0002132)
Supplement: S1 Table — (DOCX) [file pgph.0002132.s002.docx]

**S 1** Search Strategy for PubMed (Search Completed on March 3, 2023)

| 1. (Sociological factors[Title/Abstract]) AND (Multiple Chronic Conditions[Title/Abstract]) 2. (Sociological factors[MeSH Terms]) AND (Multiple Chronic Conditions[MeSH Terms]) 3. (Sociological factors[Title/Abstract]) AND (Multimorbidity [Title/Abstract]) 4. (Sociological factors[MeSH Terms]) AND (Multimorbidity [MeSH Terms]) 5. (Sociological factors[Title/Abstract]) AND (Comorbidity[Title/Abstract]) 6. (Sociological factors[MeSH Terms]) AND (Comorbidity [MeSH Terms]) 7. (Social Determinants of Health [Title/Abstract]) AND (Comorbidity[Title/Abstract]) 8. (Social Determinants of Health [MeSH Terms]) AND (Comorbidity [MeSH Terms]) 9. (Social Determinants of Health[MeSH Terms]) AND (Multimorbidity*[MeSH Terms]) 10. (Healthcare access) AND "Multiple Chronic Conditions"[ MeSH Terms] 11. (Access to healthcare) AND "Multiple Chronic Conditions"[ MeSH Terms] 12. ("Multiple Chronic Conditions"[ MeSH Terms]) AND "Social Determinants of Health"[Mesh]) AND "Health Services Accessibility"[ MeSH Terms] 13. ("Multimorbidity"[ MeSH Terms]) AND "Health Services Accessibility"[ MeSH Terms] 14. ("Health Services Accessibility"[ MeSH Terms]) AND "Comorbidity"[ MeSH Terms] 15. ("Qualitative Research"[Mesh]) AND "Multimorbidity"[ MeSH Terms]) AND "Health Services Accessibility"[ MeSH Terms] 16. (("Health Services Accessibility"[Mesh]) AND "Sociological Factors"[ MeSH Terms]) AND "Qualitative Research"[ MeSH Terms] 17. ("Qualitative Research"[ MeSH Terms]) AND "Sociological Factors"[ MeSH Terms]) AND "Multimorbidity"[ MeSH Terms]) AND "Health Services Accessibility"[ MeSH Terms] 18. ("Health Services Accessibility"[ MeSH Terms]) AND "Multimorbidity"[ MeSH Terms]) AND "Comorbidity"[ MeSH Terms]) AND "Sociological Factors"[MeSH Terms] 19. (("Comorbidity"[ MeSH Terms]) AND "Multimorbidity"[ MeSH Terms]) AND "Sociological Factors"[ MeSH Terms] 20. (Patient perspective) [All Fields] AND (Multimorbidity [Title/Abstract]) 21. (Patient experience) [All Fields] AND (Multimorbidity [Title/Abstract]) AND Access to Healthcare [All Fields] 22. (Patient experience) [All Fields] AND (Multimorbidity [Title/Abstract]) AND Access to Healthcare [All Fields] 23. (Patient experience) [All Fields] AND (Multimorbidity [Title/Abstract]) AND (Social Determinants of Health) [MeSH Terms] 24. (Patient experience) [All Fields] AND (Multimorbidity [Title/Abstract]) AND (Social Determinants of Health) AND Qualitative Research [MeSH Terms] 25. (Patient experience) [All Fields] AND (Multimorbidity [Title/Abstract]) AND Health Services Accessibility [All Fields] 26. (Multimorbidity*[MeSH Terms]) OR (Social determinants of health [MeSH Terms])) OR (Sociological factors[MeSH Terms]) OR (Patient experience) 27. (Social determinants of health [MeSH Terms]) OR (Multimorbidity*[MeSH Terms])) AND (Self-management*[MeSH Terms])) AND (qualitative research[MeSH Terms]) 28. (Sociological factors [MeSH Terms]) OR (Multimorbidity*[MeSH Terms])) AND (Self-management*[MeSH Terms])) OR (Self-care [MeSH Terms]) AND (qualitative research[MeSH Terms]) 29. (Sociological factors[Title/Abstract]) AND (Multimorbidity*[Title/Abstract])) OR (Comorbidity*[Title/Abstract]) AND (Self-care*[Title/Abstract]) OR (Self-management [Title/Abstract]) AND (qualitative research[Title/Abstract]) 30. (Professional-patient relations* [Title/Abstract]) AND (Multimorbidity*[Title/Abstract]) OR (Comorbidity*[Title/Abstract]) AND (Self-care*[Title/Abstract]) OR (Self-management [Title/Abstract]) OR (self-monitoring [Title/Abstract]) AND (qualitative research[Title/Abstract]) 31. (Sociological factors[Title/Abstract]) AND (professional-patient relations*[Title/Abstract]) AND (Grounded Theory[Title/Abstract]) AND (Multimorbidity*[Title/Abstract]) OR (comorbidity*[Title/Abstract]) AND (Self-care[Title/Abstract]) OR (self-management*[Title/Abstract]) 32. (Social determinants of health [Title/Abstract]) AND (professional-patient relations*[Title/Abstract]) AND (Phenomenology*[All Fields])) AND (Multimorbidity*[Title/Abstract]) OR (comorbidity*[Title/Abstract]) AND (Self-care[Title/Abstract]) OR (self-management*[Title/Abstract]) 33. (Social determinants of health [Title/Abstract]) AND (professional-patient relations*[Title/Abstract]) AND (Qualitative Descriptive*[All Fields]) AND (Multimorbidity*[Title/Abstract]) ) OR (comorbidity*[Title/Abstract]) AND (Self-care[Title/Abstract]) OR (self-management*[Title/Abstract]) |
| --- |
