# Supplementary material for: Sociocultural and patient-health care professional related factors influencing self-management of multiethnic patients with multimorbidities: A thematic synthesis
Source: PLOS Glob Public Health. 2023 Sep 21;3(9):e0002132. doi: 10.1371/journal.pgph.0002132 (PMC10513255; doi:10.1371/journal.pgph.0002132)
Supplement: Attachment — Submitted filename: Authors Response.docx [file pgph.0002132.s004.docx]

| **Editor’s Comments** | **Authors’ Response** |
| --- | --- |
| Please review your reference list to ensure that it is complete and correct. If you have cited papers that have been retracted, please include the rationale for doing so in the manuscript text, or remove these references and replace them with relevant current references. Any changes to the reference list should be mentioned in the rebuttal letter that accompanies your revised manuscript. If you need to cite a retracted article, indicate the article’s retracted status in the References list and also include a citation and full reference for the retraction notice. | Thank you for this reminder. We did not include any retracted articles in our review. |
| Please send a completed 'Competing Interests' statement, including any COIs declared by your co-authors. If you have no competing interests to declare, please state "The authors have declared that no competing interests exist". Otherwise please declare all competing interests beginning with twhe statement "I have read the journal's policy and the authors of this manuscript have the following competing interests:" | Thank you for this reminder. We have included a COIs statement with the submission. |
| 3. Please provide separate figure files in .tif or .eps format only and remove any figures embedded in your manuscript file. Please also ensure all files are under our size limit of 10MB. | We have provided figures in .tif format and removed the figures embedded in our manuscript. |
| **Reviewer 1** | |
| The information on gender distribution, age range, and racial/ethnic backgrounds adds to the understanding of the population under study. However, it is suggested to mention the total number of participants included in the meta-synthesis, as this would provide a clearer perspective on the sample size and generalizability of the findings. | Thank you for this excellent suggestion. We have provided the total number of patients and informal or formal caregivers interviewed in the reviewed studies.  We have added the statement:  “The total number of participants in all the reviewed studies were 660 patients with multimorbidity and 27 formal or informal caregivers”  Please note the changes at the following section.   - Page 10, under Findings; Literature Overview and Quality Rating, lines 6-7. |
| While the study highlights the need for targeted interventions to address the identified barriers, it should include a discussion or analysis of existing interventions that have been implemented in diverse contexts. | Thank you for this suggestion. While, the discussion of existing self-management interventions is important; the purpose of our review was to explore patient perceived barriers and sociocultural factors. Therefore, we cannot provide a detailed analysis of what interventions have been used across various context. However, we have added a brief description of the interventions that could be used based on previously published systematic reviews on interventions. We have added the following description.  “Health care organizations should focus on developing and implementing more targeted interventions to provide effective care to individuals with multimorbidities. Dineen-Griffin and colleagues ^14^, based on a systematic review of 58 studies, identified several interventions to promote self-management among individuals with multimorbidity. The commonly reported interventions included: knowledge and training programs, development of personalized self-care actions pans, coping and stress management programs, and medication adherence and lifestyle improvement programs. These interventions can be tailored and adapted after considering the sociocultural and patient-professional factors identified in the review, and placing mechanisms to address the negative impact of sociocultural determinants”.  Please note the changes at the following section.   - Page 21, under “Discussion”, paragraph 1. |
| Inclusion of more literature having statistical analyses, such as regression models or correlation analyses, could help determine the associations between sociocultural factors (e.g., socioeconomic status, cultural beliefs, social support) and self-management outcomes among individuals with multimorbidities. | Thank you for this comment. While, it is important to offer statistical analyses of sociocultural determinants, we did not conduct a review of quantitative studies. Our review include qualitative studies which do not report statistical analyses. However, we have noted this in areas for future research. We have added the following statement.  “We conducted a meta-synthesis of qualitative studies which, by its nature, does not offer statistical analyses of sociocultural factors affecting self-management. Therefore, it would be useful to conduct future systematic review of quantitative literature to determine the associations among various sociocultural factors and self-management”.  Please note the changes at the following section.   - Page 23, under “Limitations and Implications for Future Research”, paragraph 1, lines 6-14. |
| The most of the reviewed studies originated from from the USA (n=5) and Australia (n=4), Canada (n=3), UK (n=3), and New Zealand (n=2), followed by one each in South Africa, Ghana, the Netherlands, and Denmark, which raises concerns about the generalizability of the findings to other geographical regions and cultural contexts. Conducting a more extensive literature review to include studies from diverse regions and populations could enhance the study's generalizability and provide a more comprehensive understanding of the sociocultural and patient-health care professional factors influencing self-management among individuals with multimorbidities. | Thank you for this comment. We agree that inclusion of studies from high income countries is a major limitation. This is because we could not locate any studies for low and middle income countries. We have already noted this in our limitation.  “Most of the reviewed studies originated from the USA, Canada, and Australia. Therefore, the transferability of findings to other contexts may be limited. Further research is warranted to explore sociocultural and patient-professional factors affecting self-management of individuals with multimorbidity in low and middle income countries”.  Please note the changes at the following section.   - Page 23, under “Limitations and Implications for Future Research”, paragraph 1, lines 6-14. |
| **Reviewer 2** | |
| Most of the findings indicate that it is mostly focused on the experience of different ethnic people living in poverty, less educated, and high-income countries which should be cleared in the method section and somehow in the title. | Thank you for this comment. We agree that most of the participants belonged to diverse multiethnic groups. We have revised the title accordingly and provided brief description in the methods. Our title is already quite long so we decided to only include multiethnic in the title. Please note the changes at the following sections.   - Title - Page 10-11, under “Demographics”, lines 4-10. |
| The paper could be enriched if the authors have given more examples from low-middle-income countries. | Thank you for this comment. We agree that we did not include studies from low and middle income countries; it is because we did not find any studies from these countries except one from South Africa and one from Ghana. Therefore, unfortunately, we cannot provide analysis of barriers from low and middle income countries. We have already noted this in our limitations.  “Most of the reviewed studies originated from the USA, Canada, and Australia. Therefore, the transferability of findings to other contexts may be limited. Further research is warranted to explore sociocultural and patient-professional factors affecting self-management of individuals with multimorbidity in low and middle income countries”.  Please note the changes at the following section.   - Page 23, under “Limitations and Implications for Future Research”, paragraph 1, lines 6-14. |
| - Since the authors have discussed the measurement to minimization of the problems, I think it would be better to discuss the problems according to the context/high-income/low-income country | Thank you for this comment. As we already noted, we did not have adequate number of studies from low and middle income countries. Therefore, unfortunately, such comparative analyses cannot be undertaken. |
| There are lots of headings (excluding findings) in the paper which is sometimes confusing and I don’t think all of them are necessary | Thank you for this comment. We respectfully disagree that there are too many headings. All the headings are necessary to ensure the transparent and complete reporting of the review. Therefore, we decided to keep all the headings. |
| Literature Overview and Quality Rating and Demographics – these two paragraphs should go to the Method section rather than Findings. | Thank you for this comment. While for some readers, these findings may be better presented in the methods section; we believe that the demographics and quality rating and characteristics of the studies should be reported under findings to avoid confusion. |
| A few points are in the PDF version | Thank you for those comments. We have edit the manuscript accordingly. Please note the changes in red color throughout. |
| Please check the highlighted words or lines – I didn’t understand those well | Thank you for noting this. We have corrected some of the statements. However, one of the statement is a direct quote from the published article and therefore we are unable to change it. |
